# Supplementary material for: Herbarium specimen sequencing allows precise dating of Xanthomonas citri pv. citri diversification history
Source: Nat Commun. 2023 Jul 20;14:4306. doi: 10.1038/s41467-023-39950-z (PMC10359311; doi:10.1038/s41467-023-39950-z)
Supplement: Supplementary file 1 — Supplementary Information [file 41467_2023_39950_MOESM1_ESM.pdf]

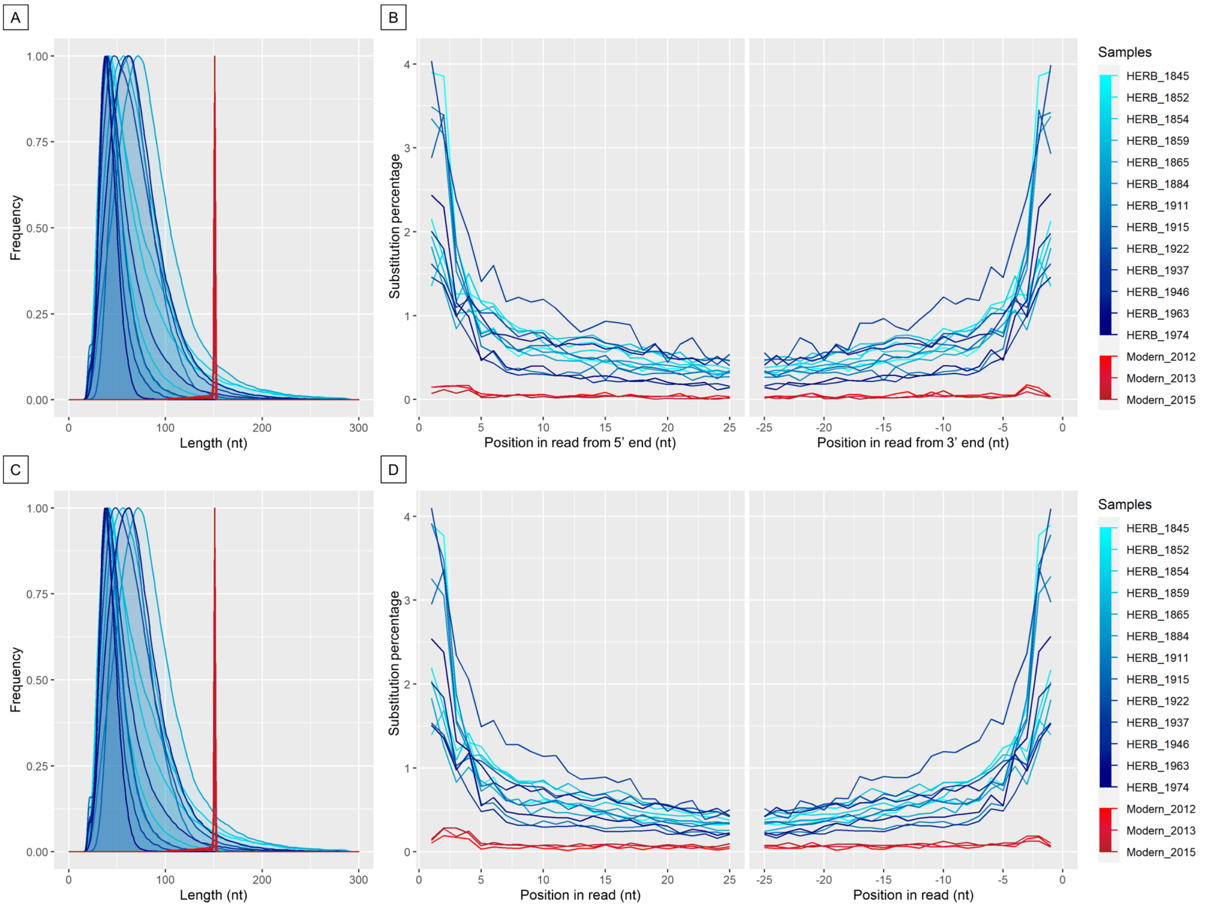

Fig S1. *Post-mortem* DNA damage patterns on reads mapping to *Xci* plasmids pXAC33 and pXAC64. (A&C) Fragment length distribution (relative frequency in arbitrary units) of reads mapping to plasmids pXAC33 and pXAC64 of the reference strain, respectively and (B&D) Substitution percentage of the first 25 nucleotides of R2 reads (5' C to T substitutions, left panel), complementary to the last 25 nucleotides of R1 reads (3' G to A substitutions, right panel)of the 13 historical genomes (blue lines, light to dark gradient from the oldest to the youngest) and three modern *Xci* strains (red lines, light to dark gradient from the oldest to the youngest) for reads mapping to plasmids pXAC33 and pXAC64 of the reference strain, respectively.

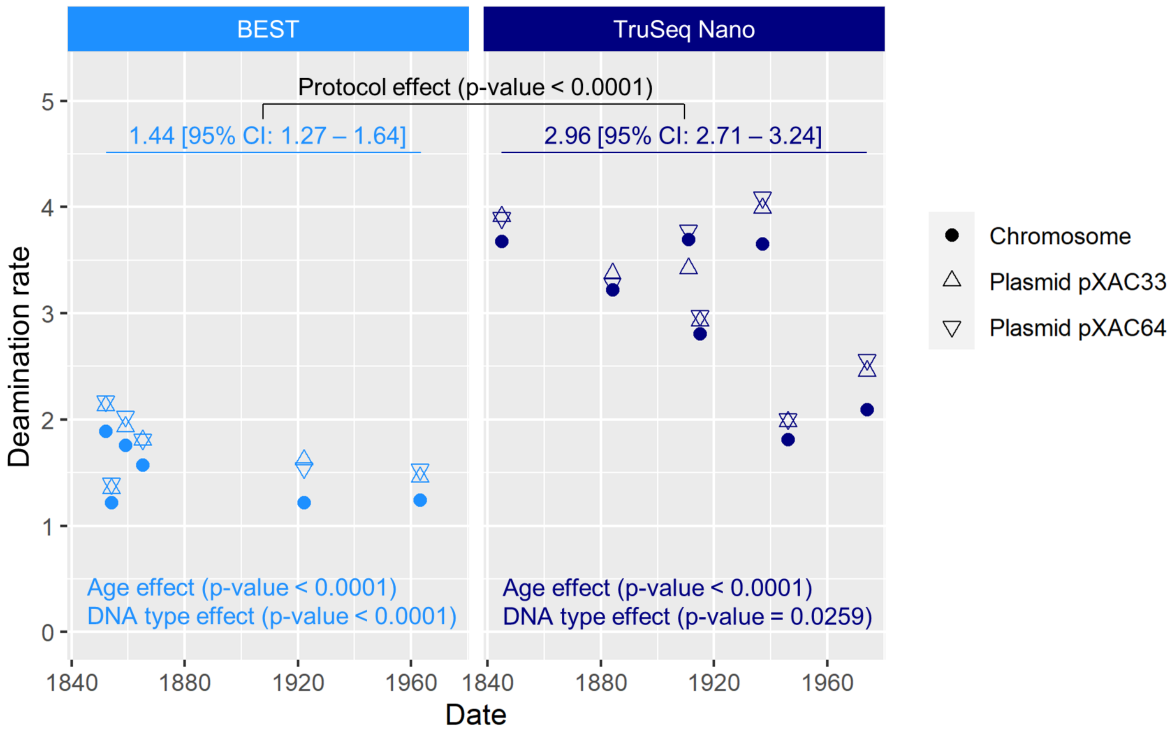

Fig S2. Terminal deamination rate (substitution percentage) from the 3' end (G to A substitutions) as a function of age of sample and DNA type for the 13 Xci genomes reconstructed from herbarium specimens. *Xci* sequencing reads displayed significantly different deamination rates, depending on the library protocol used (BEST, lightblue *vs* TruSeq Nano, darkblue; protocol effect, analysis of variance test; CI: confidence interval). Within each protocol dataset, an age effect on deamination was observed (generalized linear model test). Furthermore, reads mapping to plasmid (triangle) were significantly more deaminated than those mapping to chromosome (circle) references (DNA type effect, generalized linear mixed model test).

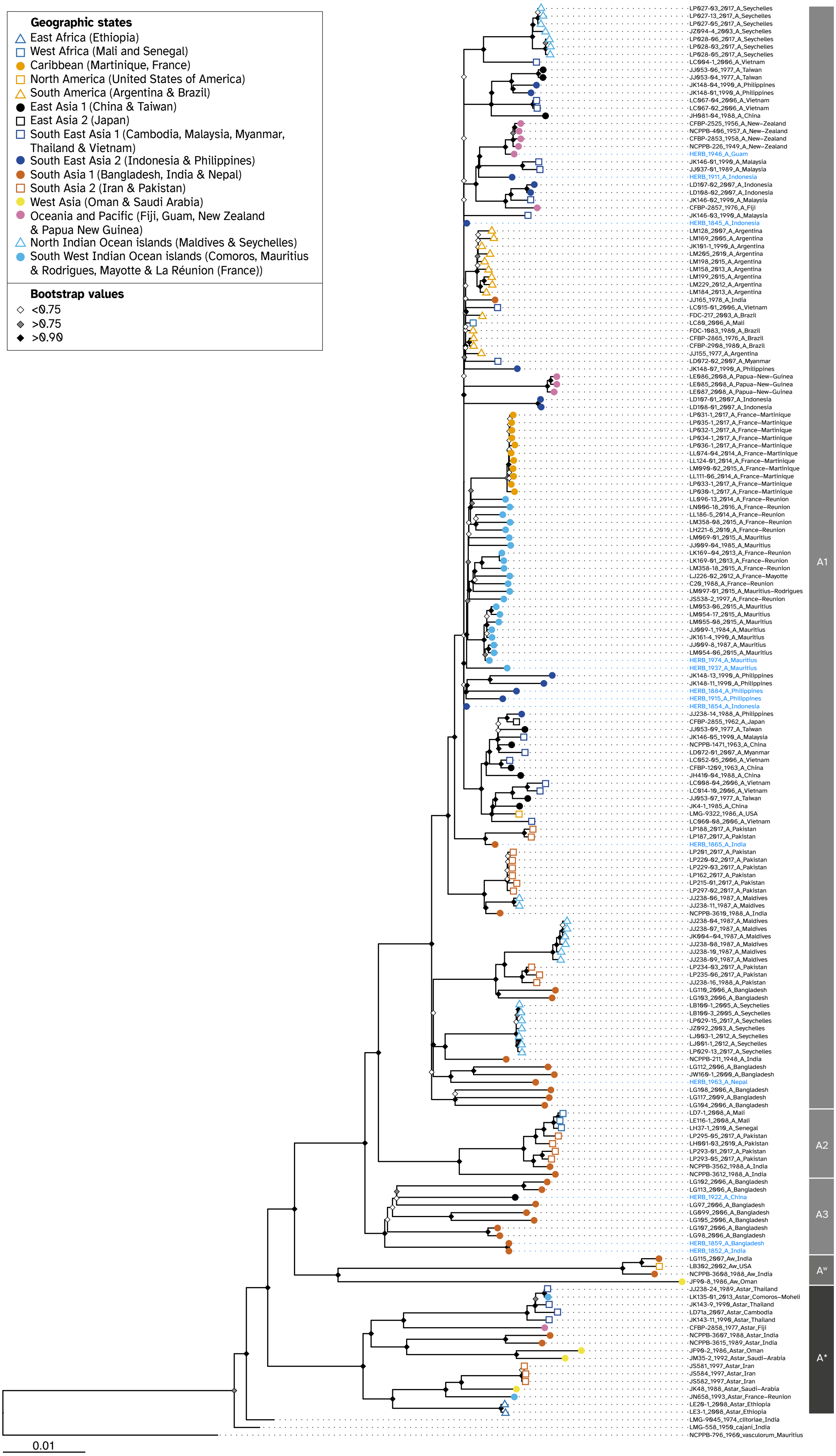
Geographic states
East Africa (Ethiopia)
West Africa (Mali and Senegal)
Caribbean (Martinique, France)
North America (United States of America)
South America (Argentina & Brazil)
East Asia 1 (China & Taiwan)
East Asia 2 (Japan)
South East Asia 1 (Cambodia, Malaysia, Myanmar, Thailand & Vietnam)
South East Asia 2 (Indonesia & Philippines)
South Asia 1 (Bangladesh, India & Nepal)
South Asia 2 (Iran & Pakistan)
West Asia (Oman & Saudi Arabia)
Oceania and Pacific (Fiji, Guam, New Zealand & Papua New Guinea)
North Indian Ocean islands (Maldives & Seychelles)
South West Indian Ocean islands (Comoros, Mauritius & Rodrigues, Mayotte & La Réunion (France))
Bootstrap values
<0.75
>0.75
>0.90
LP027-03_2017_A_Seychelles
LP027-13_2017_A_Seychelles
LP027-05_2017_A_Seychelles
JZ094-4_2003_A_Seychelles
LP028-06_2017_A_Seychelles
LP028-03_2017_A_Seychelles
LP028-05_2017_A_Seychelles
LC004-1_2006_A_Vietnam
JJ053-06_1977_A_Taiwan
JJ053-04_1977_A_Taiwan
JK148-04_1990_A_Philippines
JK148-01_1990_A_Philippines
LC067-04_2006_A_Vietnam
LC067-02_2006_A_Vietnam
JH081-04_1988_A_China
CFBP-2525_1956_A_New–Zealand
NCPPB-406_1957_A_New–Zealand
CFBP-2853_1958_A_New–Zealand
NCPPB-226_1949_A_New–Zealand
HERB_1946_A_Guam
JK146-01_1990_A_Malaysia
JJ037-01_1989_A_Malaysia
HERB_1911_A_Indonesia
LD107-02_2007_A_Indonesia
LD108-02_2007_A_Indonesia
JK146-02_1990_A_Malaysia
CFBP-2857_1976_A_Fiji
JK146-03_1990_A_Malaysia
HERB_1845_A_Indonesia
LM128_2007_A_Argentina
LM169_2005_A_Argentina
JK101-1_1990_A_Argentina
LM205_2010_A_Argentina
LM198_2015_A_Argentina
LM158_2013_A_Argentina
LM199_2015_A_Argentina
LM229_2012_A_Argentina
LM184_2013_A_Argentina
JJ165_1978_A_India
LC015-01_2006_A_Vietnam
FDC-217_2003_A_Brazil
LC80_2006_A_Mali
FDC-1083_1980_A_Brazil
CFBP-2865_1976_A_Brazil
CFBP-2908_1980_A_Brazil
JJ155_1977_A_Argentina
LD072-02_2007_A_Myanmar
JK148-07_1990_A_Philippines
LE086_2008_A_Papua–New–Guinea
LE085_2008_A_Papua–New–Guinea
LE087_2008_A_Papua–New–Guinea
LD107-01_2007_A_Indonesia
LD108-01_2007_A_Indonesia
LP031-1_2017_A_France–Martinique
LP035-1_2017_A_France–Martinique
LP032-1_2017_A_France–Martinique
LP034-1_2017_A_France–Martinique
LP036-1_2017_A_France–Martinique
LL074-04_2014_A_France–Martinique
LL124-01_2014_A_France–Martinique
LM090-02_2015_A_France–Martinique
LL111-06_2014_A_France–Martinique
LP033-1_2017_A_France–Martinique
LP030-1_2017_A_France–Martinique
LL096-13_2014_A_France–Reunion
LN006-18_2016_A_France–Reunion
LL186-5_2014_A_France–Reunion
LM358-08_2015_A_France–Reunion
LH221-6_2010_A_France–Reunion
LM069-01_2015_A_Mauritius
JJ009-04_1985_A_Mauritius
LK169–04_2013_A_France–Reunion
LK169-01_2013_A_France–Reunion
LM358-18_2015_A_France–Reunion
LJ226-02_2012_A_France–Mayotte
C20_1988_A_France–Reunion
LM097-01_2015_A_Mauritius–Rodrigues
JS538-2_1997_A_France–Reunion
LM053-06_2015_A_Mauritius
LM054-17_2015_A_Mauritius
LM055-08_2015_A_Mauritius
JJ009-1_1984_A_Mauritius
JK161-4_1990_A_Mauritius
JJ009-8_1987_A_Mauritius
LM054-06_2015_A_Mauritius
HERB_1974_A_Mauritius
HERB_1937_A_Mauritius
JK148-13_1990_A_Philippines
JK148-11_1990_A_Philippines
HERB_1884_A_Philippines
HERB_1915_A_Philippines
HERB_1854_A_Indonesia
JJ238-14_1988_A_Philippines
CFBP-2855_1962_A_Japan
JJ053-09_1977_A_Taiwan
JK146-05_1990_A_Malaysia
NCPPB-1471_1963_A_China
LD072-01_2007_A_Myanmar
LC052-05_2006_A_Vietnam
CFBP-1209_1963_A_China
JH410-04_1988_A_China
LC008-04_2006_A_Vietnam
LC014-10_2006_A_Vietnam
JJ053-07_1977_A_Taiwan
JK4-1_1985_A_China
LMG-9322_1986_A_USA
LC060-08_2006_A_Vietnam
LP188_2017_A_Pakistan
LP187_2017_A_Pakistan
HERB_1865_A_India
LP201_2017_A_Pakistan
LP220-02_2017_A_Pakistan
LP229-03_2017_A_Pakistan
LP162_2017_A_Pakistan
LP215-01_2017_A_Pakistan
LP297-02_2017_A_Pakistan
JJ238-06_1987_A_Maldives
JJ238-11_1987_A_Maldives
NCPPB-3610_1988_A_India
JJ238-04_1987_A_Maldives
JJ238-07_1987_A_Maldives
JK004–04_1987_A_Maldives
JJ238-08_1987_A_Maldives
JJ238-10_1987_A_Maldives
JJ238-09_1987_A_Maldives
LP234-03_2017_A_Pakistan
LP235-06_2017_A_Pakistan
JJ238-16_1988_A_Pakistan
LG110_2006_A_Bangladesh
LG103_2006_A_Bangladesh
LB100-1_2005_A_Seychelles
LB100-3_2005_A_Seychelles
LP029-15_2017_A_Seychelles
JZ092_2003_A_Seychelles
LJ003-1_2012_A_Seychelles
LJ001-1_2012_A_Seychelles
LP029-13_2017_A_Seychelles
NCPPB-211_1948_A_India
LG112_2006_A_Bangladesh
JW160-1_2000_A_Bangladesh
HERB_1963_A_Nepal
LG108_2006_A_Bangladesh
LG117_2009_A_Bangladesh
LG104_2006_A_Bangladesh
LD7-1_2008_A_Mali
LE116-1_2008_A_Mali
LH37-1_2010_A_Senegal
LP295-05_2017_A_Pakistan
LH001-03_2010_A_Pakistan
LP293-01_2017_A_Pakistan
LP293-05_2017_A_Pakistan
NCPPB-3562_1988_A_India
NCPPB-3612_1988_A_India
LG102_2006_A_Bangladesh
LG113_2006_A_Bangladesh
HERB_1922_A_China
LG97_2006_A_Bangladesh
LG099_2006_A_Bangladesh
LG105_2006_A_Bangladesh
LG107_2006_A_Bangladesh
LG98_2006_A_Bangladesh
HERB_1859_A_Bangladesh
HERB_1852_A_India
LG115_2007_Aw_India
LB302_2002_Aw_USA
NCPPB-3608_1988_Aw_India
JF90-8_1986_Aw_Oman
JJ238-24_1989_Astar_Thailand
LK135-01_2013_Astar_Comoros–Moheli
JK143-9_1990_Astar_Thailand
LD71a_2007_Astar_Cambodia
JK143-11_1990_Astar_Thailand
CFBP-2858_1977_Astar_Fiji
NCPPB-3607_1988_Astar_India
NCPPB-3615_1989_Astar_India
JF90-2_1986_Astar_Oman
JM35-2_1992_Astar_Saudi–Arabia
JS581_1997_Astar_Iran
JS584_1997_Astar_Iran
JS582_1997_Astar_Iran
JK48_1988_Astar_Saudi–Arabia
JN658_1993_Astar_France–Reunion
LE20-1_2008_Astar_Ethiopia
LE3-1_2008_Astar_Ethiopia
LMG-9045_1974_clitoriae_India
LMG-558_1950_cajani_India
NCPPB-796_1960_vasculorum_Mauritius
A1
A2
A3
Aw
A*
0.01

Fig S3. Maximum Likelihood (ML) phylogenetic tree of historical and modern *Xci* genomes. ML tree including 13 historical specimens (labelled in blue) and 171 modern strains (black) built from 13,007 recombination-free SNPs. *Xanthomonas axonopodis* pv. *vasculorum* NCPPB-796 isolated in 1960 from Mauritius (GenBank accession number: GCF_013177355.1) was used to root the tree. Node values correspond to bootstrap values calculated on 1,000 iterations. Strain labels include strain name, collection year, pathotype and country of origin; branch tips are colored according to the sample's geographic origin (see Materials and Methods for details). Pathotypes and lineages are indicated to the right.

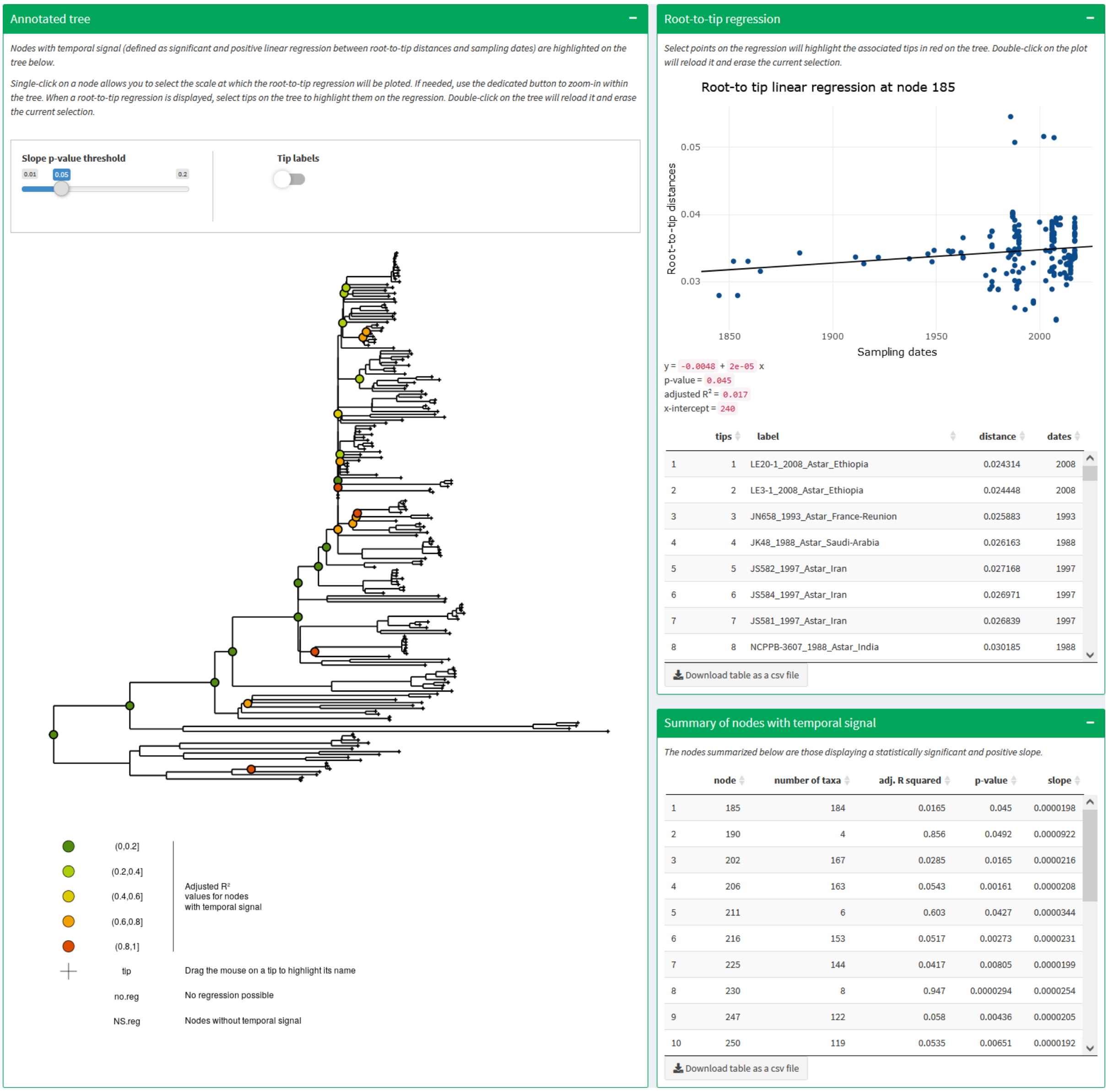

Fig S4. Root-to-tip regression temporal test visualized on online tool PhyloStemS. Nodes with temporal signal, *i.e.* nodes at which root-to-tip linear regression yielded a statistically significant and positive slope, are highlighted with colors scaling to R2 value. The tool is accessible online (https://pvbmt-apps.cirad.fr/apps/phylostems/).

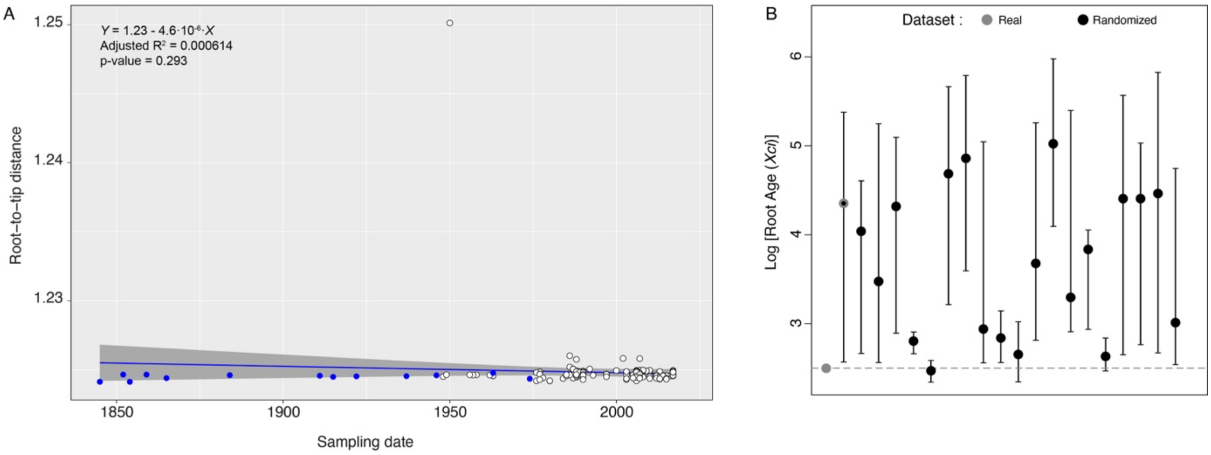

Fig S5. Root-to-tip regression and date-randomization temporal test results when performed on the dataset including outgroups. (A) Root-to-tip linear regression line plotted in black with historical genomes (*n* = 13, blue dots) and modern ones (*n* = 174, white dots with black line). Grey area (error band) indicates 95% confidence interval. Associated values are the linear regression equation, adjusted R^2 and p-value obtained from a two-sided Student test under the null hypothesis of a slope equal to zero, with (*n-1*) degrees of freedom. (B) Evaluating temporal signal in the dataset by date-randomization test showed overlap between the age of the root estimated from the real dataset (grey) vs 20 date-randomized datasets (black). Black points represent median values and error bars represent 95% Highest Posterior Density intervals computed from n= 10,000 iterations.

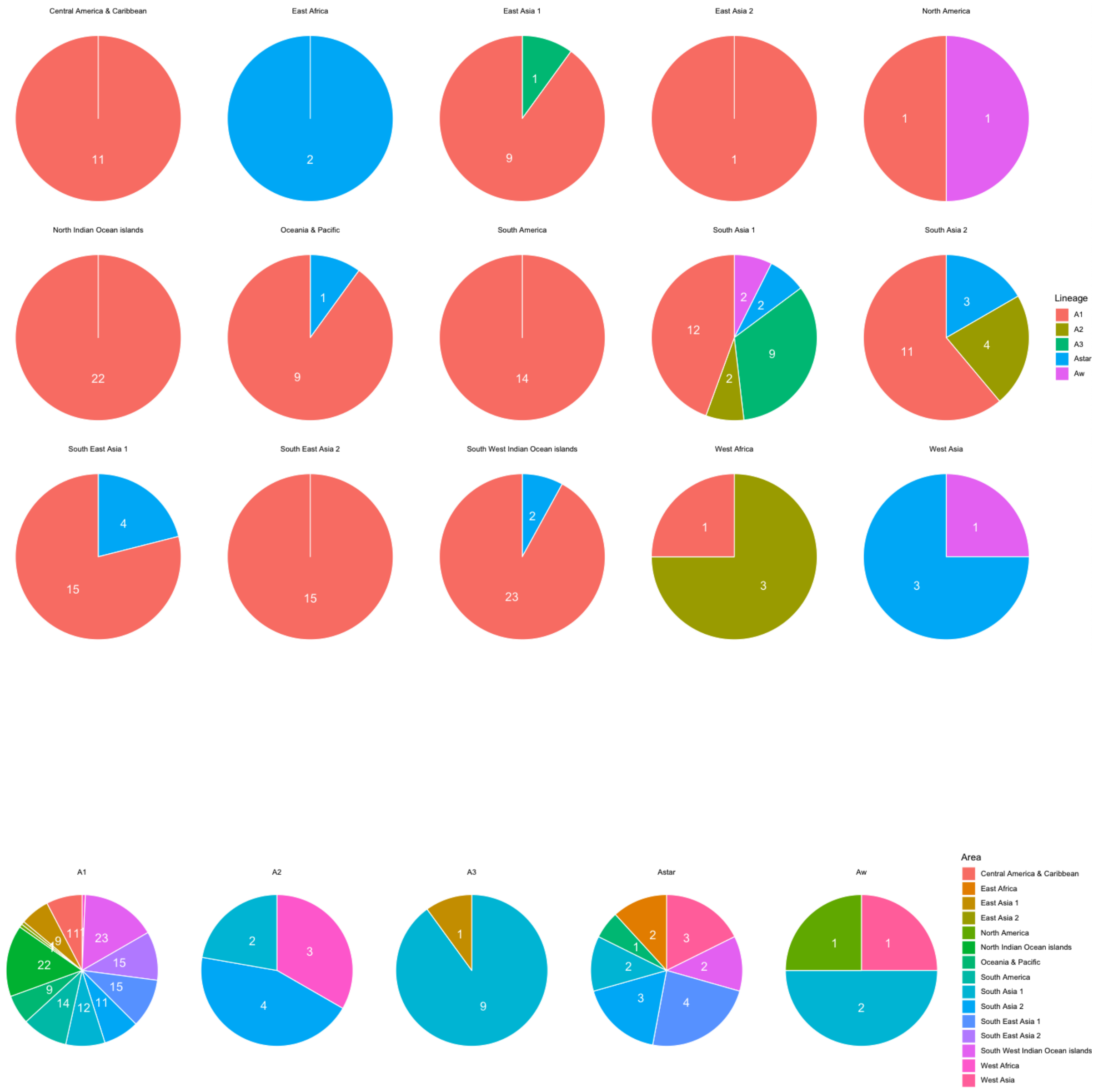

Fig S6. Illustration of the geographic clustering observed within the phylogenetic tree. Pie charts displaying the fraction of samples belonging to each lineage within each geographic region (top), and conversely (bottom).

Table S1. Summary of mapping, depth, coverage and damage statistics for reads of the 13 historic *Xci* mapping to plasmids pXAC33 and pXAC64 of the reference strain. SD: standard deviation; nt: nucleotides.

		***Xci* DNA (%)**		**Mean depth**		**Coverage at 1X (%)**		**Insert length (mean ± SD in nt)**		**Deamination rate at terminal position (%)**	
**ID**	**Protocol**	**pXAC33**	**pXAC64**	**pXAC33**	**pXAC64**	**pXAC33**	**pXAC64**	**pXAC33**	**pXAC64**	**pXAC33**	**pXAC64**
HERB_1845	TruSeq Nano	0.36	0.48	122.2	120.4	93.3	95.7	52.2±22.8	51.9±22.6	3.91	3.90
HERB_1884	TruSeq Nano	0.35	0.54	92.1	94.6	89.8	93.2	48.4±17.0	48.4±16.9	3.38	3.28
HERB_1911	TruSeq Nano	0.06	0.08	69.2	65.2	90.2	90.2	69.7±21.9	69.4±21.8	3.42	3.78
HERB_1915	TruSeq Nano	0.16	0.24	66.2	65.8	88	92.9	48.5±17.1	48.7±17.1	2.93	2.98
HERB_1937	TruSeq Nano	0.03	0.04	22.6	17.9	82.9	88.5	44.7±13.8	44.6±13.6	3.99	4.09
HERB_1946	TruSeq Nano	0.23	0.32	114.9	108.1	94.7	93.2	58.3±28.1	58.4±28.2	1.98	2.01
HERB_1974	TruSeq Nano	0.12	0.14	42.6	25.5	82.5	49.7	41.5±9.5	41.6±9.5	2.46	2.56
HERB_1852	BEST	0.17	0.23	130.3	117.9	95.9	96.5	70.4±41.4	69.7±41.1	2.13	2.17
HERB_1854	BEST	0.39	0.54	114	108.6	95.2	97.1	72.0±39.6	72.3±39.7	1.35	1.40
HERB_1859	BEST	0.16	0.22	84.1	75.4	94.2	95.3	71.7±31.6	71.4±31.4	1.93	2.03
HERB_1865	BEST	0.13	0.20	95.1	101.5	77.9	96.6	85.1±36.8	85.1±36.7	1.81	1.81
HERB_1922	BEST	0.60	0.99	99.6	128.7	76.5	94.5	67.7±29.2	67.2±28.5	1.62	1.54
HERB_1963	BEST	0.55	0.67	106.8	86.2	94.3	96	73.2±31.0	73.0±30.9	1.46	1.53

Table S2. Recombining regions among 184 historical or modern *Xci* strains. Positions are indicated relatively to the reference genome of strain IAPAR 306.

Starting position	Ending position	Length
3,095,443	3,119,514	24,071
3,799,050	3,805,347	6,297
4,257,584	4,630,760	373,176
4,959,140	4,988,729	29,589
