# Supplementary material for: Herbarium specimen sequencing allows precise dating of Xanthomonas citri pv. citri diversification history
Source: Nat Commun. 2023 Jul 20;14:4306. doi: 10.1038/s41467-023-39950-z (PMC10359311; doi:10.1038/s41467-023-39950-z)
Supplement: Supplementary file 3 — Description of Additional Supplementary Files [file 41467_2023_39950_MOESM3_ESM.pdf]

Description of Additional Supplementary Files:

Supplementary data 1: General characteristics of the historical and modern strains of the study. Labels consist in strain ID, year of collection, *Xci* pathotype (or other *Xanthomonas* pathovar: vasculorum= *Xanthomonas axonopodis* pv. *vasculorum*; clitoriae= *Xanthomonas citri* pv. *clitoriae*; cajani= *Xanthomonas citri* pv. *cajani*) and country of origin. Geographical origin areas are described in the Methods.

Supplementary data 2: List and presence status of 144 pathogenicityassociated genes investigated among 184 historical or modern *Xci* strains. A gene was considered present if its coding sequence reached at least 75% coverage of the corresponding CDS in the organism strain. *Xci*: *Xanthomonas citri* pv. *citri*; *Xe: Xanthomonas euvesicatoria; Xcc: Xanthomonas campestris* pv. *campestris; Xoc: Xanthomonas oryzae* pv. *oryzicola; Xoo: Xanthomonas oryzae* pv. *oryzae*; T3E: Type III effector; T3SS: Type III secretion system. Most genes were identified and characterized in the papers listed at the end of the table.

Supplementary data 3: Coverage (in %) of the 10 pathogenicity-associated genes of variable presence investigated among 184 historical or modern *Xci* strains.
