# Supplementary material for: Herbarium specimen sequencing allows precise dating of Xanthomonas citri pv. citri diversification history
Source: Nat Commun. 2023 Jul 20;14:4306. doi: 10.1038/s41467-023-39950-z (PMC10359311; doi:10.1038/s41467-023-39950-z)
Supplement: Supplementary file 7 — Reporting Summary [file 41467_2023_39950_MOESM7_ESM.pdf]

nature portfolio

Corresponding author(s): Adrien RIEUX

Last updated by author(s): May 31, 2023

Reporting Summary

Nature Portfolio wishes to improve the reproducibility of the work that we publish. This form provides structure for consistency and transparency in reporting. For further information on Nature Portfolio policies, see our Editorial Policies and the Editorial Policy Checklist.

Statistics

For all statistical analyses, confirm that the following items are present in the figure legend, table legend, main text, or Methods section.

n/a	Confirmed	
☐	☒	The exact sample size (*n*) for each experimental group/condition, given as a discrete number and unit of measurement
☐	☒	A statement on whether measurements were taken from distinct samples or whether the same sample was measured repeatedly
☐	☒	The statistical test(s) used AND whether they are one- or two-sided<br>*Only common tests should be described solely by name; describe more complex techniques in the Methods section.*
☐	☒	A description of all covariates tested
☐	☒	A description of any assumptions or corrections, such as tests of normality and adjustment for multiple comparisons
☐	☒	A full description of the statistical parameters including central tendency (e.g. means) or other basic estimates (e.g. regression coefficient) AND variation (e.g. standard deviation) or associated estimates of uncertainty (e.g. confidence intervals)
☐	☒	For null hypothesis testing, the test statistic (e.g. *F*, *t*, *r*) with confidence intervals, effect sizes, degrees of freedom and *P* value noted<br>*Give P values as exact values whenever suitable.*
☐	☒	For Bayesian analysis, information on the choice of priors and Markov chain Monte Carlo settings
☐	☒	For hierarchical and complex designs, identification of the appropriate level for tests and full reporting of outcomes
☐	☒	Estimates of effect sizes (e.g. Cohen's *d*, Pearson's *r*), indicating how they were calculated

Our web collection on statistics for biologists contains articles on many of the points above.

Software and code

Policy information about availability of computer code

Data collection	All datasets analyzed in this study were generated using Microsoft Excel v16.71 and/or R v4.1.1 softwares.
Data analysis	The following softwares were used to analyse the data presented in this article: R v4.1.1, BBMap 37.92, AdapterRemoval 2.2.2, mapDamage 2.2.1, BWA-aln 0.7.15,Bowtie 2, picardtools 2.7.0, BEDTools genomecov 2.24.0, GATK v4.2 UnifiedGenotyper, RAxML 8.2.4, Phylostems 1.0, BEAST 1.8.4, Tracer 1.7.1, TreeAnnotator v1.0, ClonalFrameML, Trimmomatic 0.36, BamUtil 1.0.14, TipDatingBeast v1.0 R package, stats R package v1.0 & ggtree v1.0 R package.

For manuscripts utilizing custom algorithms or software that are central to the research but not yet described in published literature, software must be made available to editors and reviewers. We strongly encourage code deposition in a community repository (e.g. GitHub). See the Nature Portfolio guidelines for submitting code & software for further information.

Data

Policy information about availability of data

All manuscripts must include a data availability statement. This statement should provide the following information, where applicable:

- Accession codes, unique identifiers, or web links for publicly available datasets
- A description of any restrictions on data availability
- For clinical datasets or third party data, please ensure that the statement adheres to our policy

The authors confirm that all data used in this study are fully available without restriction. Both historical and modern raw reads were deposited to the Sequence Read Archive (under accession numbers listed in Table S1). Accession numbers of any previously published data used in this study are also listed in Table S1.

Research involving human participants, their data, or biological material

Policy information about studies with human participants or human data. See also policy information about sex, gender (identity/presentation), and sexual orientation and race, ethnicity and racism.

Reporting on sex and gender	not applicable
Reporting on race, ethnicity, or other socially relevant groupings	not applicable
Population characteristics	not applicable
Recruitment	not applicable
Ethics oversight	not applicable

Note that full information on the approval of the study protocol must also be provided in the manuscript.

Field-specific reporting

Please select the one below that is the best fit for your research. If you are not sure, read the appropriate sections before making your selection.

☐ Life sciences ☐ Behavioural & social sciences ☒ Ecological, evolutionary & environmental sciences

For a reference copy of the document with all sections, see nature.com/documents/nr-reporting-summary-flat.pdf

Ecological, evolutionary & environmental sciences study design

All studies must disclose on these points even when the disclosure is negative.

Study description	In this study, we reconstructed 13 historical genomes of the bacterial crop pathogen Xanthomonas citri pv. citri (Xci) from infected citrus herbarium specimens dating back to 1845. We compared them to a large set (N=171) of modern genomes to reconstruct their phylogenetic relationships, pathogeny-associated genes content and estimate several evolutionary parameters. Each bacterial genome generated during the course of this study represent an unique and independent unit.
Research sample	A group of 13 historical Citrus specimens sampled from different herbaria worldwide and a group of 57 modern Xci strains obtained from living bacterial collections worldwide. In addition to the sampling strategy indicated below, the historical specimens described in this study where those for which good quality DNA sequences were obtained. All the specimens generated belongs to the species Xanthomonas citri pv citri.
Sampling strategy	Historical samples displaying typical Asiatic citrus canker lesions were sampled. Those were chosen as the oldest available from Asia, the supposed geographic origin of Xci, as well as from Oceania and the Southwest Indian Ocean. Modern strains were chosen to get a good representation of both the genetic and geographical diversity of the pathogen.
Data collection	A.R, PC, LG and NB collected the different historical samples. Citrus specimens displaying typical citrus canker lesions were sampled in herbaria using gloves and sterile equipment and brought back to CIRAD laboratory in individual envelopes. Collection of any plant material used in this study complies with institutional, national, and international guidelines. Permission to collect and analyze each historical specimen included in this study was provided by the herbarium institutions (and their curators) from which they were sampled.
Timing and spatial scale	Historical specimens were sampled between 2017 and 2021 in various herbaria. They were initially collected between 1845 and 1974 from various locations in Asia and South-West Indian Ocean Islands.
Data exclusions	No data were excluded from analysis.

Reproducibility	We could not repeat any historical samples treatment due to the destructive nature of our investigations and the low quantity of material available.
Randomization	Samples were allocated into groups either from their source: historical (from herbarium specimens) vs modern (from living bacteria) or their geographical origin.
Blinding	Blinding was used during lab work from DNA extraction to sequencing. Both samples and blanks were randomly labelled to avoid manipulations bias.

Did the study involve field work? ☐ Yes ☒ No

Reporting for specific materials, systems and methods

We require information from authors about some types of materials, experimental systems and methods used in many studies. Here, indicate whether each material, system or method listed is relevant to your study. If you are not sure if a list item applies to your research, read the appropriate section before selecting a response.

Materials & experimental systems

n/a	Involved in the study	
☒	☐	Antibodies
☒	☐	Eukaryotic cell lines
☒	☐	Palaeontology and archaeology
☒	☐	Animals and other organisms
☒	☐	Clinical data
☒	☐	Dual use research of concern
☐	☒	Plants

Methods

n/a	Involved in the study	
☒	☐	ChIP-seq
☒	☐	Flow cytometry
☒	☐	MRI-based neuroimaging

Dual use research of concern

Policy information about dual use research of concern

Hazards

Could the accidental, deliberate or reckless misuse of agents or technologies generated in the work, or the application of information presented in the manuscript, pose a threat to:

No	Yes	
☒	☐	Public health
☒	☐	National security
☒	☐	Crops and/or livestock
☒	☐	Ecosystems
☒	☐	Any other significant area

Experiments of concern

Does the work involve any of these experiments of concern:

No	Yes	
☒	☐	Demonstrate how to render a vaccine ineffective
☒	☐	Confer resistance to therapeutically useful antibiotics or antiviral agents
☒	☐	Enhance the virulence of a pathogen or render a nonpathogen virulent
☒	☐	Increase transmissibility of a pathogen
☒	☐	Alter the host range of a pathogen
☒	☐	Enable evasion of diagnostic/detection modalities
☒	☐	Enable the weaponization of a biological agent or toxin
☒	☐	Any other potentially harmful combination of experiments and agents
